# Erythropoietin Mitigates Diabetic Nephropathy by Restoring PINK1/Parkin-Mediated Mitophagy

**DOI:** 10.3389/fphar.2022.883057

**Published:** 2022-05-17

**Authors:** Xinyao Yi, Wenhui Yan, Tingli Guo, Na Liu, Zhuanzhuan Wang, Jia Shang, Xiaotong Wei, Xin Cui, Yuzhuo Sun, Shuting Ren, Lina Chen

**Affiliations:** ^1^ Department of Pharmacology, School of Basic Medical Sciences, Health Science Center, Xi’an Jiaotong University, Xi’an, China; ^2^ Department of Phathology, School of Basic Medical Sciences, Health Science Center, Xi’an Jiaotong University, Xi’an, China; ^3^ Key Laboratory of Environment and Genes Related to Diseases, Ministry of Education, Xi’an Jiaotong University, Xi’an, China

**Keywords:** EPO, Pink1/parkin, LC3, mitophagy, diabetic nephropathy

## Abstract

Diabetic nephropathy (DN), one of the most detrimental microvascular complications of diabetes, is the leading cause of end-stage renal disease. The pathogenesis of DN is complicated, including hemodynamic changes, inflammatory response, oxidative stress, among others. Recently, many studies have demonstrated that mitophagy, especially PINK1/Parkin-mediated mitophagy, plays an important role in the pathogenesis of DN. Erythropoietin (EPO), a glycoprotein hormone mainly secreted by the kidney, regulates the production of erythrocytes. This research intends to explore the beneficial effects of EPO on DN and investigate related mechanisms. In in *vitro* experiments, we found that EPO promoted autophagic flux and alleviated mitochondrial dysfunction in terms of mitochondrial fragmentation, elevated mitochondrial ROS as well as the loss of mitochondrial potential, and lowered the apoptosis level in high-glucose-treated mesangial cells. Moreover, EPO increased protein expressions of PINK1 and Parkin, enhanced the co-localization of LC3 with mitochondria, Parkin with mitochondria as well as LC3 with Parkin, and increased the number of GFP-LC3 puncta, resulting in increased level of PINK1/Parkin-mediated mitophagy in mesangial cells. The knockdown of PINK1 abrogated the effect of EPO on mitophagy. In addition, *in vivo* experiments demonstrated that EPO attenuated renal injury, reduced oxidative stress, and promoted expressions of genes related to PINK1/Parkin-mediated mitophagy in the kidneys of DN mice. In summary, these results suggest that PINK1/Parkin-mediated mitophagy is involved in the development of DN and EPO mitigates DN by restoring PINK1/Parkin-mediated mitophagy.

## Introduction

Diabetes mellitus (DM), which is characterized by hyperglycemia, is one of the most hazardous chronic metabolic diseases. According to the International Diabetes Federation, the number of DM patients worldwide will increase to 629 million by 2045 (Saxena et al., 2019). DM can induce many severe diabetic complications, including microvascular and macrovascular complications. Diabetic nephropathy (DN) is a kind of microvascular complication of DM, it can further induce 40–50% of end-stage renal diseases (Haller et al., 2017). The main pathological features of DN include proteinuria, decreased glomerular filtration rate and progressively abnormal renal functions (Zoja et al., 2020). Although the pathogenesis of DN is very complicated, multiple studies have shown that abnormal mitochondrial functions and inhibited mitophagy play important roles in the development of DN (Huang et al., 2016; Wei and Szeto, 2019).

The process of autophagy, which depends on lysosomes, is dedicated to specifically remove harmful or excessive cellular components such as protein aggregates or damaged organelles in cells (Martini-Stoica et al., 2016). Autophagy plays a key role in the maintenance of cellular structure and functions. Therefore, the changes of autophagy contribute to the pathogenesis of many diseases, such as aging, cancer, neurodegenerative diseases and DM (Chun and Kim, 2018). Mitophagy, a kind of selective autophagy, can particularly remove abnormal and even damaged mitochondria, which is mainly mediated by the PINK1/Parkin pathway (Zhu et al., 2013). PINK1/Parkin-mediated mitophagy is inhibited in DN, together with dysfunctional mitochondrial dynamics and functions (Higgins and Coughlan, 2014). A study demonstrated that the mRNA expressions of PINK1 and Parkin as well as the level of mitophagy in glomerular mesangial cells of DN patients were significantly decreased (Czajka et al., 2015). In addition, another study showed that renal cells, which were exposed to high glucose (HG), displayed abnormal mitochondrial dynamics and inhibited PINK1/Parkin-mediated mitophagy (Chen et al., 2018).

Erythropoietin (EPO), a glycoprotein hormone mainly secreted by the kidney, regulates the production of erythrocytes (Nishimura et al., 2018). Clinically, EPO is mainly used for the treatment of anemia (Fishbane et al., 2018). Studies have shown that EPO has protective effects on multiple organs and prevents tissue injury during inflammation and ischemia (Eren et al., 2016; Cui et al., 2017; Lee et al., 2017; Zhang et al., 2019). Importantly EPO can alleviate diseases through promoting autophagic flux and enhancing autophagy (Jang et al., 2016; Wang et al., 2018; Zhong et al., 2020). Moreover, EPO has been reported to improve the decreased mitochondrial membrane potential, reduce the level of mitochondrial ROS, and restore dysfunctional mitochondrial morphology and biosynthesis (Zhu et al., 2008; Cui et al., 2017). Of note, the concentration of EPO is relatively low in the serum of patients with diabetes or kidney diseases (Mercadal et al., 2012; Fujita et al., 2019). According to the previous research, EPO can activate autophagy to produce renoprotective effects. Therefore, we aim to explore the role of EPO in DN and whether EPO could restore PINK1/Parkin-mediated mitophagy in order to mitigate DN.

## Materials and Methods

### Animals

Male C57BL/6 mice weighing 16–20 g were purchased from Medical Experimental Animal Center of Xi’an Jiaotong University. All mice were housed in a temperature-controlled room (22–24°C) with a 12 h light/dark cycle and have free access to diets and water. Mice were classified into Control group (*n* = 8) and high-fat-diet group (HFD, *n* = 20) randomly according to body weight. Control mice were fed with normal chow diets, while HFD mice were fed with high-fat diets. After 8 weeks, following fasting for 12 h, mice in the Control group and HFD group were intraperitoneally injected with citrate buffer and 60 mg/kg body weight streptozotocin (STZ, Sigma-Aldrich, United States) respectively for three consecutive days. Three days after that, fasting blood glucose (FBG) was examined after fasting. DM model is successfully achieved if the FBG of the mice in the HFD group is 15–25 mmol/L for two consecutive days. Then the DM mice were randomly divided into Model group (*n* = 8) and EPO group (*n* = 8). The mice in EPO group were subcutaneously injected with 500 IU/kg body weight recombinant human EPO (3SBIOINC., China) 3 times a week, while the mice in Control group and Model group were subcutaneously injected with normal saline. The body weight and FBG of each mouse were measured weekly. After the administration for 6 weeks, mice were sacrificed and the serum and kidney tissues were obtained for subsequent experiments. All animal experimental procedures were approved by the Institutional Animals Care and Use Committee at Xi’an Jiaotong University and in accordance with the National Institutes of Health Guide for Care and Use of Laboratory Animals.

### Cell Culture and Treatment

Human glomerular mesangial cells were kindly provided by Prof. Ren (School of Basic Medical Sciences, Xi’an Jiaotong University, China). Mesangial cells were cultured in low glucose Dulbecco’s Modified Eagle’s Medium (DMEM, Hyclone, United States) supplemented with 10% fetal bovine serum (Biological Industries, Israel), 100 U/ml penicillin plus 100 μg/ml streptomycin (Solarbio, China) under 5% CO2 at 37°C. Cells were treated with low glucose (Control, 5.5 mM) and high glucose (HG, 30 mM) with or without EPO (20 IU/ml) for 48 h. For all experiments, cells were cultured in serum-free medium for 12 h before HG and EPO administration.

### Biochemical Measurements

For detecting biochemical changes of kidney tissues and serum, renal tissues were homogenized in phosphate-buffered saline (PBS). The levels of MDA and SOD activity of tissue homogenates as well as the levels of Creatinine (CRE) and Blood Urea Nitrogen (BUN) were measured by assay kits (Beyotime, China).

### Histopathological Studies

Pathological changes of kidney tissues were detected as described previously (Chen et al., 2018). In brief, paraffin-embedded renal tissue sections went through de-waxing in xylene and rehydration in decreasing grades of alcohol, and then tissue sections were stained by hematoxylin-eosin (HE) stain, Masson stain and periodic acid-Schiff stain according to manufacturer’s instructions. Results were observed by a light microscrope (Olympus, Japan).

### Western Blotting

Total protein of renal tissues and glomerular mesangial cells was extracted by RIPA lysis buffer supplemented with protease inhibitors (Solarbio, China). Protein concentrations were determined using the BCA Protein Kit (Solarbio, China). All samples were boiled at 95°C for 5 min. Equal amounts of proteins were separated in 8–12% SDS-PAGE gels and then transferred onto PVDF membranes (Millipore, United States). After blocked with 5% non-fat milk for 2 h, the membranes were incubated with various primary antibodies overnight at 4°C, namely rabbit anti-LC3B (1:1,500, GeneTex, United States), rabbit anti-P62 (1:1,000, Proteintech, China), rabbit anti-PINK1 (1:1,000, Abcam, United Kingdom), mouse anti-Parkin (1:200, Santa Cruz Biotechnology, United States), rabbit anti-Drp-1 (1:1,000, Cell Signaling Technology, United States), rabbit anti-Mfn-2 (1:1,000, Cell Signaling Technology, United States). And then the membranes were washed 3 times with Tris-buffered saline containing Tween-20 for around 15 min and subsequently incubated with corresponding secondary antibodies for 2 h. After washing 3 times with TBST for around 15 min, the bands were detected by using ECL reagents (Abbkine, United States) and their density was quantified by using ImageJ software.

### Real-Time PCR

Total RNA was isolated from renal tissues using RNA isolation kit (Takara, Japan). RNA was reverse transcribed into cDNA using qPCR-RT Master mix kit (Takara, Japan). Finally, cDNA was analyzed by real-time PCR using the SYBR Green qPCR Mix (Takara, Japan). Relative expression of target mRNA was calculated based on the 2^−ΔΔCt^ comparative method and normalized to that of the internal standard (*β*-actin). The primers are listed in Table 1.

**TABLE 1:** | Sequences of primers for real-time PCR.

Gene	Forward primer	Reverse primer
LC3B	CCC​ACC​AAG​ATC​CCA​GTG​AT	TTG​GTT​AGC​ATT​GAG​CTG​CAA
P62	AGA​ATG​TGG​GGG​AGA​GTG​TG	TCT​GGG​GTA​GTG​GGT​GTC​AG
PINK1	GCT​TGC​CAA​TCC​CTT​CTA​TG	CTC​TCG​CTG​GAG​CAG​TGA​C
Parkin	TGT​GAC​CTG​GAA​CAA​CAG​AGT​A	TCA​GGT​CCA​CTC​GTG​TCA​A
*β*-actin	TGT​GAC​GTT​GAC​ATC​CGT​AA	GCT​AGG​AGC​CAG​AGC​AGT​AA

### Detection of Mitochondrial ROS and Mitochondrial Transmembrane Potential

Mesangial cells were seeded in 12-well plates and exposed to different treatments. As for the measurement of mitochondrial ROS, the treated cells were incubated with MitoSOX Red (Invitrogen, United States) and Mito-tracker Green (Beyotime, China) for 20 min according to manufacturer’s instructions. In terms of the detection of mitochondrial transmembrane potential, the treated cells were incubated with JC-1 working solution (Beyotime, China) for 30 min, and then the cells were washed by JC-1 washing buffer for 3 times. After that, fluorescence images were observed by a fluorescence microscope (Olympus, Japan).

### GFP-LC3 Lentivirus Transfection

In order to detect the level of mitophagy, the colocalization of mitochondria with autophagosome was achieved by GFP-LC3 lentivirus transfection and mitochondrial staining as reported previously (Jiang et al., 2020). Briefly, mesangial cells were infected with GFP-LC3 lentivirus (Genechem, China) at a suitable MOI of 50 in accordance with manufacturer’s instructions. After 72 h of transfection, the positive cells were screened out by the treatment of puromycin (5 ug/ml). The viable cells, after being exposed to HG and EPO for 48 h, were used to measure the colocalization of mitochondria with autophagosome by staining mitochondria through Mito-tracker Red (Beyotime, China). Fluorescence images were observed by a fluorescence microscope (Olympus, Japan).

### Immunofluorescence Staining

Mesangial cells were cultured in 12-well plates with coverslips. After receiving various treatments for 48 h, cells were fixed with 4% paraformaldehyde at room temperature for 20 min and washed 3 times with PBS for 15 min. Then cells were permeabilized with 0.1% Triton X-100 for 30 min at 4°C. After being blocked with goat serum for 2 h, cells were incubated with primary antibodies overnight at 4°C, namely rabbit anti-LC3B (GeneTex, United States), rabbit anti-PINK1 (Abcam, United Kingdom), mouse anti-Parkin (Santa Cruz Biotechnology, United States). After that, cells were washed 3 times for 15 min and incubated with fluorescent secondary antibody (Zhuangzhi, China) for 2 h in the darkroom at room temperature. Eventually, the nuclei of cells were stained with DAPI and images were captured by a fluorescence microscope (Olympus, Japan).

### RNA Interference

PINK1 siRNA and relevant scrambled siRNA were purchased from GenePharm (Shanghai, China). Mesangial cells at 30–40% confluence, which were seeded in 6-well plates, were transfected with scrambled siRNA or PINK1 siRNA using lipofectamine 2000 according to the manufacturer’s instructions.

### Measurement of Cell Apoptosis

Cell apoptosis was detected as previously described (Tsai et al., 2020). Mesangial cells were seeded in 6-well plates. After being exposed to different treatments for 48 h, treated cells were incubated with Annexin V-FITC and PI solution (Beyotime, China) for 15 min in the darkroom at room temperature. The level of cell apoptosis was evaluated by flow cytometry (ACEA Biosciences, United States).

### Statistics

All data were obtained from at least three independent experiments and were presented as mean ± SEM. Results were subjected to a one-way analysis of variance (ANOVA) followed by least significant difference (LSD) test when comparing multiple groups by IBM SPSS 22.0. Values of *p* < 0.05 were considered significant.

## Results

### Erythropoietin Reversed High Glucose-Induced the Blockage of Autophagic Flux in Mesangial Cells

**FIGURE 1:**
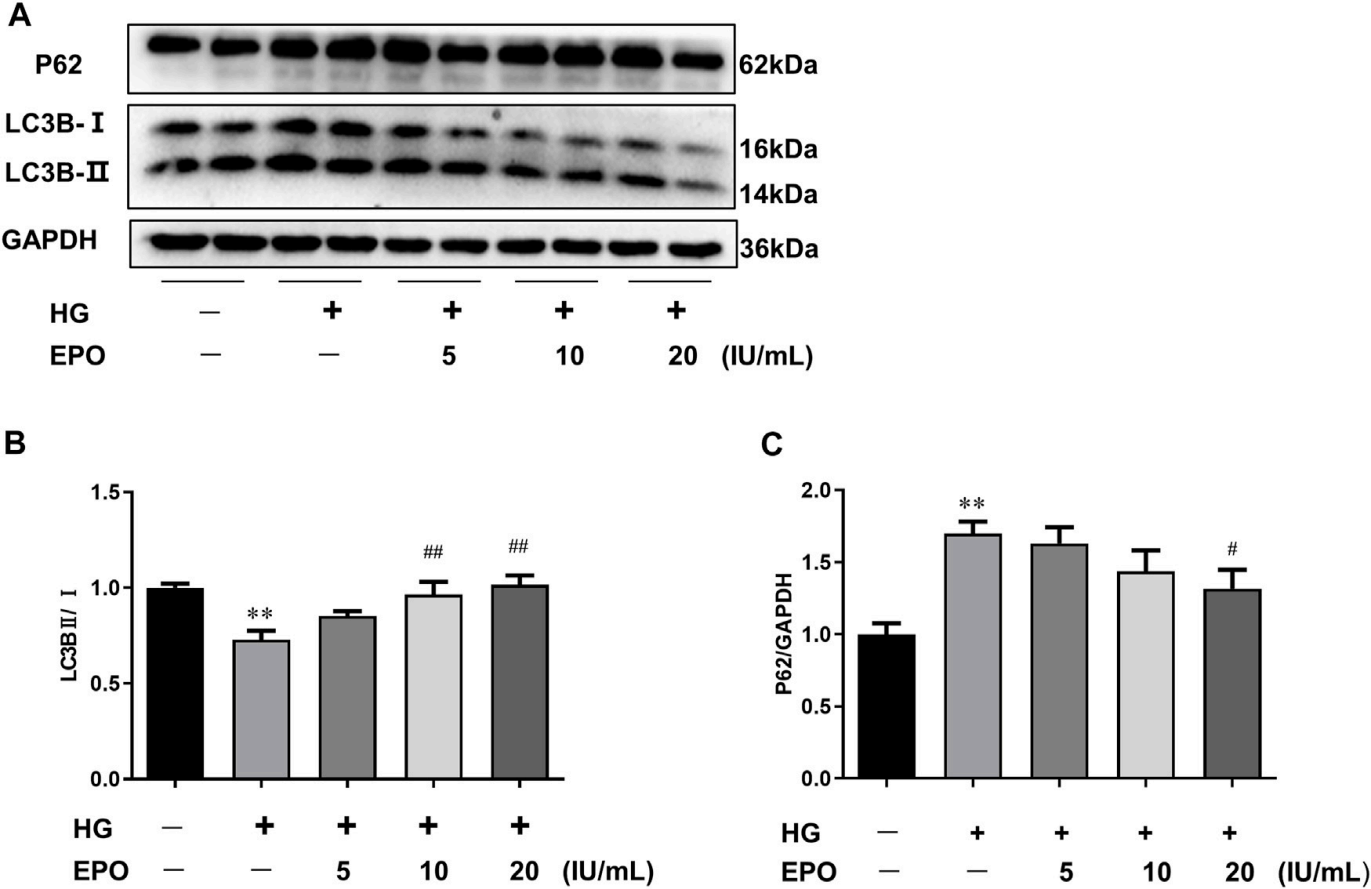
EPO reversed HG-induced the blockage of autophagic flux in mesangial cells. **(A)** Representative immunoblots and quantification of **(B)** LC3B and **(C)** P62. Values are presented as mean ± SEM, *n* = 3. **p < 0.01 vs. Control group; ^#^ *p* < 0.05, ^##^ *p* < 0.01 vs. HG group.

Autophagy and the functions of mesangial cells are associated with the development of DN, and autophagic flux serves as an important index of the level of autophagy. So HG-injured mesangial cells were used as the *in vitro* DN model and key proteins related to autophagic flux were measured. As shown in Figure 1, compared with the control group, HG significantly reduced the ratio of LC3B-Ⅱ/LC3B-Ⅰ and increased P62 protein expression, resulting in the blockage of autophagic flux. However, EPO increased the ratio of LC3B-Ⅱ/LC3B-Ⅰ and decreased the level of P62 in a dose-dependent manner (Figure 1), thereby alleviating the blockage of autophagic flux in HG-injured mesangial cells. Therefore, we choose 20 IU/ml EPO treatment to conduct next experiments.

### Erythropoietin Alleviated Mitochondrial Dysfunction and Apoptosis in Mesangial Cells Exposed to High Glucose

**FIGURE 2:**
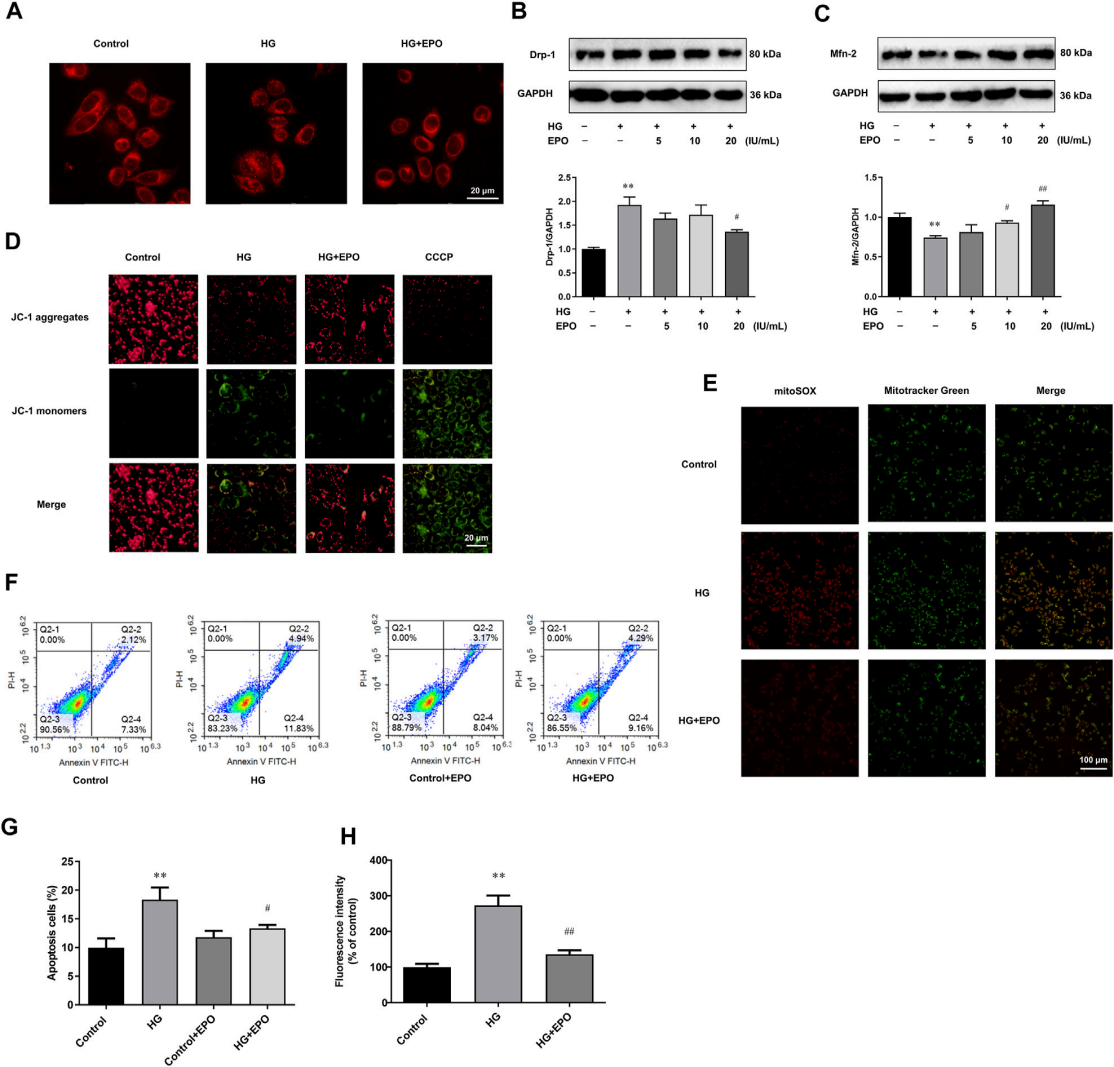
EPO alleviated mitochondrial dysfunction and apoptosis in mesangial cells exposed to HG. **(A)** Representative images of mesangial cells stained with Mitotracker-Red. Representative immunoblots and quantification of **(B)** Drp-1 and **(C)** Mfn-2. Representative images of mesangial cells stained with **(D)** JC-1 and **(E)** MitoSOX as well as **(G)** quantification of the level of mitochondrial ROS. **(F)** Flow cytometry analysis and **(H)** quantitative analysis of cell apoptosis. Values are presented as mean ± SEM, *n* = 3. **p < 0.01 vs. Control group; ^#^ *p* < 0.05, ^##^ *p* < 0.01 vs. HG group.

HG was reported to aggravate mitochondrial malfunction, as displayed by mitochondrial fragmentation as well as abnormal mitochondria membrane potential and mitochondrial ROS levels, and induce a higher level of apoptosis in DN models (Jiang et al., 2020). Consistently, compared with Control group, mesangial cells exposed to HG had a lot of fragmented mitochondria (Figure 2A), along with the up-regulated Drp-1 (Figure 2B) and down-regulated Mfn-2 protein levels (Figure 2C) which were indispensable for mitochondrial fission. Furthermore, HG induced the loss of mitochondrial membrane potential (Figure 2D), promoted the ectopic production of mitochondrial ROS (Figures 2E,G), and increased the apoptosis level (Figures 2F,H) in mesangial cells, leading to mitochondrial dysfunction. However, EPO partly restored the mitochondrial morphology as well as the loss of mitochondria membrane potential, and significantly decreased the level of mitochondrial ROS and apoptosis in HG-treated mesangial cells compared with HG group.

### PINK1/Parkin-Mediated Mitophagy was Involved in the Protective Effects of Erythropoietin on Mesangial Cells Exposed to High Glucose

**FIGURE 3:**
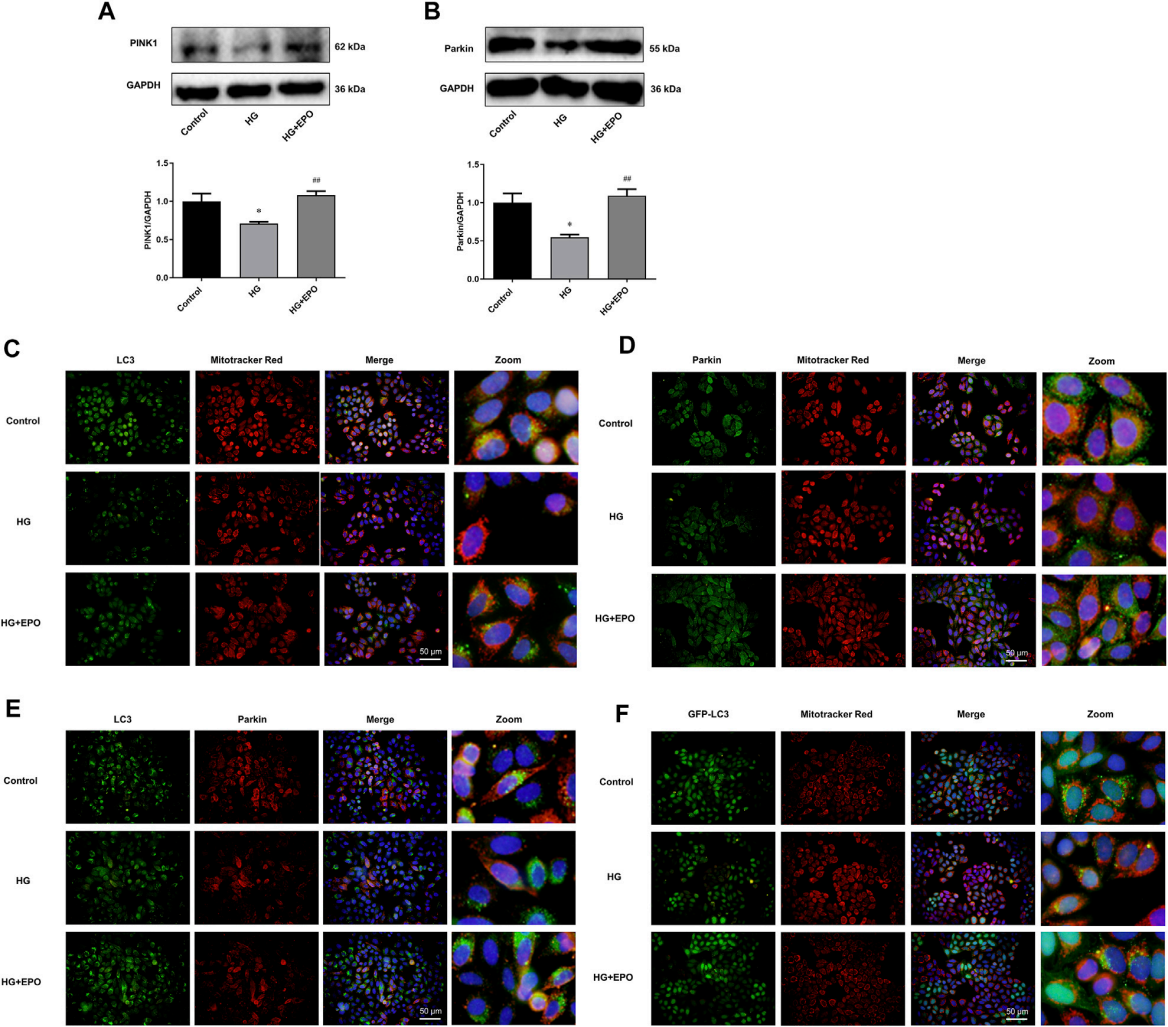
PINK1/Parkin-mediated mitophagy was involved in the protective effects of EPO on mesangial cells exposed to HG. Representative immunoblots and quantification of **(A)** PINK1 and **(B)** Parkin. Immunofluorescence images of colocalization of **(C)** LC3 with mitochondria, **(D)** Parkin with mitochondria and **(E)** LC3 with Parkin as well as the **(F)** colocalization of GFP-LC3 with mitochondria. Values are presented as mean ± SEM, *n* = 3. *p < 0.05 vs. Control group; ^#^ *p* < 0.05 vs. HG group.

In order to explore the underlying mechanism, we then identified whether the protective effects of EPO attribute to the rescue of PINK1/Parkin-mediated mitophagy in mesangial cells. Mitophagy, especially PINK1/Parkin-mediated mitophagy, has been demonstrated to play an important role in DN. Many studies have shown that decreased level of PINK1/Parkin-mediated mitophagy is involved in the development of DN (Li et al., 2017; Jiang et al., 2020). Accordingly, our results showed that HG significantly decreased the protein expression of PINK1 (Figure 3A) and Parkin (Figure 3B) compared with the control group, indicating reduced level of PINK1/Parkin-mediated mitophagy, while EPO reversed the expression of PINK1 and Parkin and promoted PINK1/Parkin-mediated mitophagy. In PINK1/Parkin-mediated mitophagy, the interaction of LC3 and mitochondria and the translocation of Parkin from cytoplasm to mitochondria are essential. Compared with the control group, HG decreased the co-localization of LC3 with mitochondria (Figure 3C), Parkin with mitochondria (Figure 3D) as well as LC3 with Parkin (Figure 3E), thereby inhibiting the translocation of LC3 to mitochondria and the translocation of Parkin from cytoplasm to mitochondria. Also, reduced number of GFP-LC3 puncta was observed in HG group (Figure 5F), suggesting a decreased level of PINK1/Parkin-mediated mitophagy. In contrast, EPO increased the co-localization of LC3 with mitochondria (Figure 3C), Parkin with mitochondria (Figure 3D) and LC3 with Parkin (Figure 3E) as well as the number of GFP-LC3 puncta (Figure 3F), indicating that PINK1/Parkin-mediated mitophagy was involved in the effect of EPO on HG-injured mesangial cells. These results demonstrated that HG inhibited PINK1/Parkin-mediated mitophagy in mesangial cells, while EPO restored this change.

### PINK1 Knockdown Partly Abrogated the Effect of Erythropoietin on the Level of Mitophagy

**FIGURE 4:**
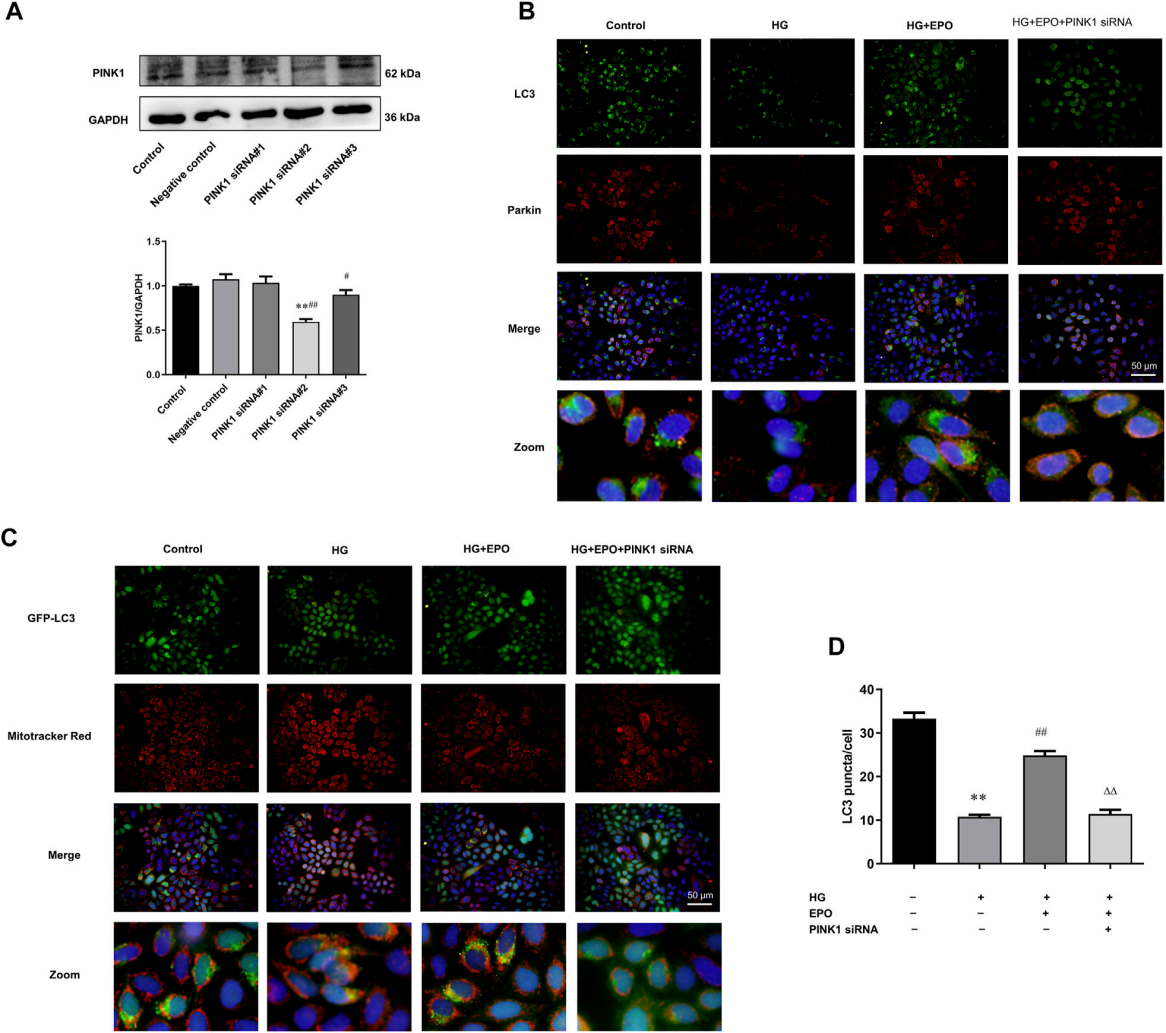
PINK1 knockdown partly abrogated the effect of EPO on the level of mitophagy. **(A)** Representative immunoblots and quantification of PINK1 when mesangial cells were treated PINK1 siRNA. Immunofluorescence images of the **(B)** colocalization of LC3 with Parkin as well as the **(C)** colocalization of GFP-LC3 with mitochondria and **(D)** quantification of the number of GFP-LC3 puncta. Values are presented as mean ± SEM, *n* = 3. **p < 0.01 vs. Control group; ^##^ *p* < 0.01 vs. HG group; ^ΔΔ^ *p* < 0.01 vs. EPO group.

In order to confirm the critical role of PINK1/Parkin-mediated mitophagy in HG-injured mesangial cells, we further used PINK1 siRNA#2 to knockdown the expression of PINK1 in mesangial cells. The knockdown efficiency of PINK1 was verified by Western blotting (Figure 4A). Again, compared with the control group, HG decreased the co-localization of LC3 and Parkin and significantly reduced the number of GFP-LC3 puncta, and EPO treatment alleviated these changes (Figures 4B-D). However, the knockdown of PINK1 partly abrogated the effects of EPO on the enhanced co-localization of LC3 with Parkin and increased number of GFP-LC3 puncta (Figures 4B,C), demonstrating that the protective effects of EPO was dependent on PINK/Parkin-mediated mitophagy.

### Erythropoietin Protected Renal Functions and Morphological Characteristics of Diabetic Nephropathy Mice

**FIGURE 5:**
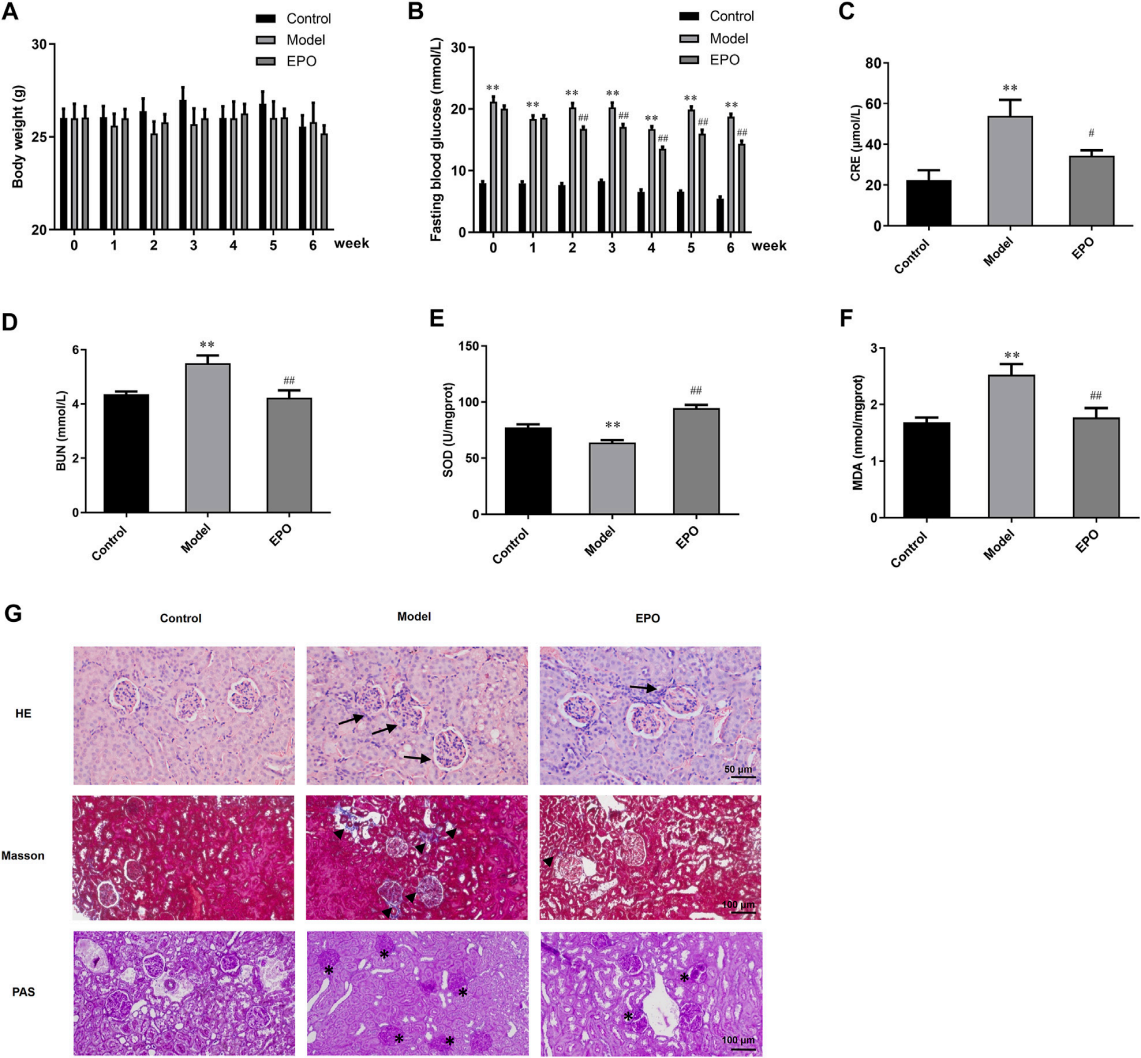
EPO protected renal functions and morphological characteristics of DN mice. **(A)** The changes of body weight, **(B)** FBG levels, **(C)** serum creatinine, **(D)** blood urea nitrogen, **(E)** SOD activity, MDA, and **(G)** histological staining of kidney tissues in terms of HE, Masson and PAS staining. Arrows indicate the abnormal glomerular structure, arrowheads indicated the fibrosis deposition and asterisks indicate the deposition of mesangial matrix. Values are presented as mean ± SEM, for **(A,B)**, *n* = 8; for **(C,D)**, *n* = 3; for **(E,F)**, *n* = 5. **p < 0.01 vs. Control group; ^#^ *p* < 0.05, ^##^ *p* < 0.01 vs. Model group.

*In vitro* experiments indicated that EPO mitigated HG-injured mesangial cells. We then explored whether EPO alleviated DN *in vivo* experiments by establishing the DN mouse model through high-fat diet combined with intraperitoneally injection with STZ. During the administration of EPO or normal saline in three different groups for 6 weeks, we examined the body weight and FBG of mice every week. As shown in Figure 5A, there was no significant difference in the body weight between these three groups in the whole 6 weeks. In terms of FBG, mice of the Model group had a significant higher level of FBG than that of the Control group, while EPO significantly decreased the FBG levels of the Model mice (Figure 5B). Moreover, compared with mice in Control group, mice in the Model group had increased levels of serum creatinine (Figure 5C), blood urea nitrogen (Figure 5D), MDA (Figure 5F) and reduced SOD activity (Figure 5E), indicating decreased renal functions and increased oxidative stress of kidney tissues. However, EPO significantly reversed these parameters mentioned above (Figures 5C–F), thereby improving renal functions of the Model mice. Subsequently, we used histological staining, including HE, Masson and PAS staining, to examine kidney morphology, collagen deposition and basement membrane thickness respectively. As shown in Figure 5G, abnormal glomerular structure, renal fibrosis and matrix accumulation were observed in kidney tissues of mice in Model group compared with the mice of the control group, while EPO administration alleviated these histological changes.

### Erythropoietin Restored PINK1/Parkin-Mediated Mitophagy in Kidney Tissues of Diabetic Nephropathy Mice

**FIGURE 6:**
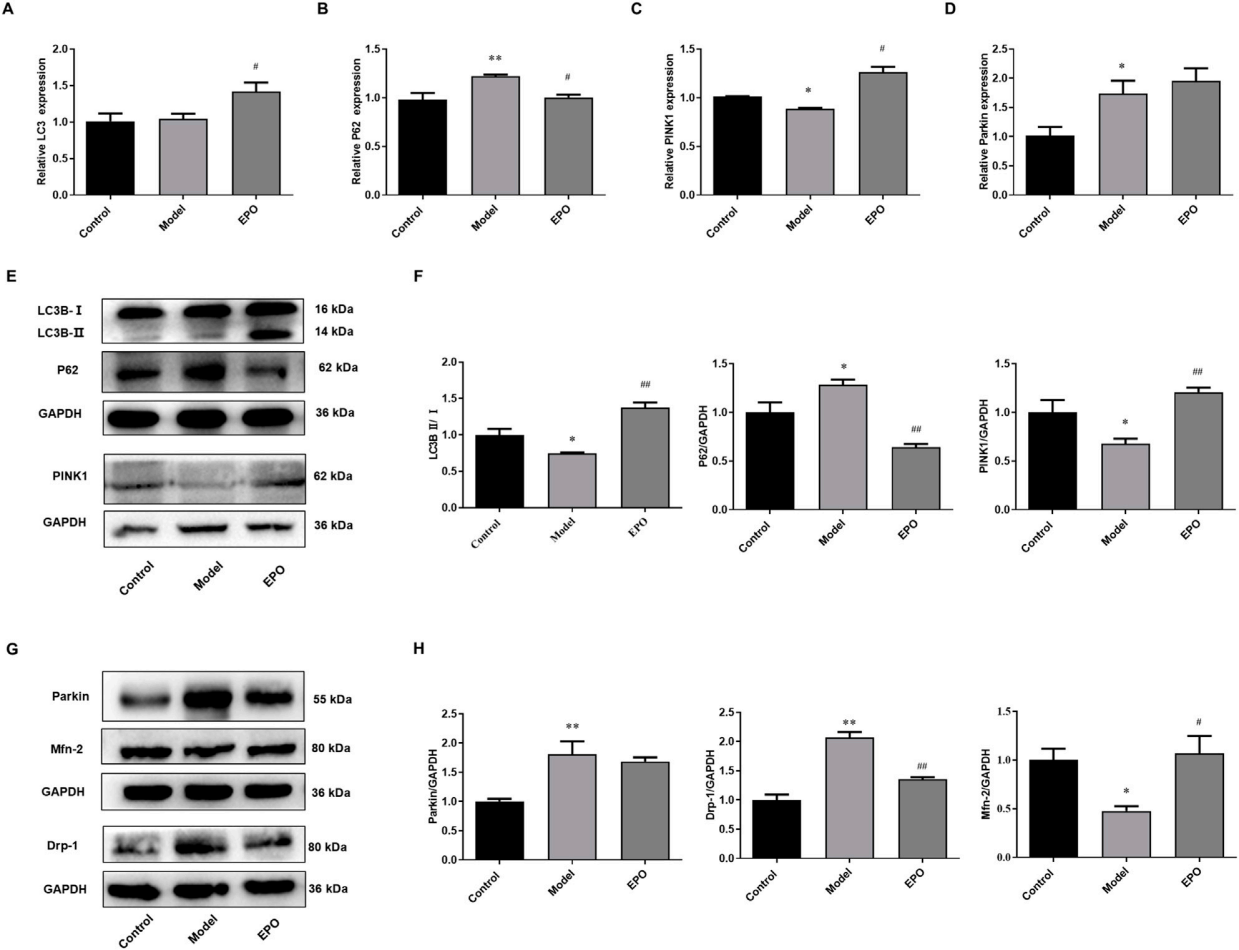
EPO activated PINK1/Parkin-mediated mitophagy in kidney tissues of DN mice. The changes of **(A)** LC3 mRNA expression, **(B)** P62 mRNA expression, **(C)** PINK1 mRNA expression, **(D)** Parkin mRNA expression, **(E)** the ratio of LC3B-II/LC3B-I, P62 protein expression and PINK1 protein expression, **(G)** Parkin protein expression, Drp-1 protein expression and Mfn-2 protein expression. **(F)** Relative quantification of LC3B-II/LC3B-I, P62 and PINK1, **(H)** Relative quantification of Parkin, Drp-1 and Mfn-2. Values are presented as mean ± SEM, n = 3. *P < 0.05, **p < 0.01 vs. Control group; #*p* < 0.05, ##*p* < 0.01 vs. Model group.

According to the *in vitro* results, the protective effects of EPO on mesangial cells exposed to HG were associated with PINK1/Parkin-mediated mitophagy. So we further detected the mRNA and protein levels of genes in PINK1/Parkin-mediated mitophagy in the kidney tissues. Compared with mice in the control group, the ratio of LC3B-II/LC3B-I (Figures 6E,F), the mRNA and protein levels of PINK1 (Figures 6C,E,F), and Mfn-2 protein level (Figures 6G,H) in the kidney tissues of mice of Model group were significantly decreased, while P62 and Drp-1 were significantly increased in terms of the mRNA and protein levels (Figures 6B,E–H), resulting in the blockage of autophagic flux and inhibited level of PINK1/Parkin-mediated mitophagy. However, there was no difference in the mRNA expression of LC3 in these two groups (Figure 6A), and the mRNA and protein levels of Parkin were elevated in the model group (Figures 6D,G,H). Of note, EPO administration partly reversed these changes as indicated by the significantly increased ratio of LC3B-II/LC3B-I (Figures 6E,F) as well as expressions of PINK1 (Figures 6C,E,F) and Mfn-2 (Figures 6G,H), and decreased expressions of P62 (Figures 6B,E,F) and Drp-1 (Figures 6G,H). These results suggested that EPO could mitigate the blockage of autophagic flux and improve the level of PINK1/Parkin-mediated mitophagy.

## Discussion

Mitochondrial dysfunction and inhibited PINK1/Parkin-mediated mitophagy play important roles in the pathogenesis of DN. Many studies have shown that protecting mitochondrial morphology and functions and enhancing PINK1/Parkin-mediated mitophagy can alleviate DN (Huang et al., 2016; Li et al., 2017; Sun et al., 2019; Wei and Szeto, 2019). Mitochondria morphology always changes in a dynamic process, including fission and fusion. Mitochondrial fission is mainly regulated by Drp-1, and the knockdown of Drp-1 protein leads to excessive mitochondrial fusion, resulting in the formation of abnormal large mitochondria. Mitochondrial fusion includes the fusion of the outer mitochondrial membrane and the inner membrane. The outer membrane fusion is mainly regulated by Mfn1/2 (Hamacher-Brady and Brady, 2016). When mitochondrial fusion is inhibited, mitochondria continue to divide and produce excessive ROS, resulting in the opening of mitochondrial permeability transition pore, the release of pro-apoptotic factors, and the loss of mitochondrial membrane potential (Fan et al., 2019). Our results also proved that in the mice of Model group, the expression of Drp-1 in kidney tissues was significantly increased, while the expression of Mfn-2 was significantly decreased, indicating excessive mitochondrial fission of the kidney tissues. We also found that the level of oxidative stress in the kidney tissue of Model mice was increased. Moreover, HG promoted mitochondrial fission, decreased the mitochondrial membrane potential and increased the level of mitochondrial ROS in glomerular mesangial cells.

PINK1/Parkin-mediated mitophagy can specifically remove damaged mitochondria, thereby inhibiting mitochondrial ROS and cell apoptosis. In DN, autophagic flux is blocked and PINK1/Parkin-mediated mitophagy is inhibited (Czajka et al., 2015; Koch et al., 2020). When activating PINK1/Parkin-mediated mitophagy, the abnormal mitochondrial morphology and functions, increased ROS and apoptosis levels can be prevented in DN (Sun et al., 2019). In the present study, we found that EPO promoted the autophagy flux in the kidney tissue of Model mice and increased the expression of PINK1 and Parkin. However, the expression of Parkin in the kidney tissues of Model mice was also increased significantly. Parkin mainly exists in the cytoplasm. With the initiation of mitophagy, PINK1 will recruit Parkin from the cytoplasm to the outer mitochondrial membrane, so the expression of Parkin in the mitochondria can better reflect the level of PINK1/Parkin-mediated mitophagy. In this study, we just detected the total Parkin protein expression in the kidney tissue of Model mice, so this may lead to different results. In mesangial cells exposed by HG, we found that EPO also enhanced PINK/Parkin-mediated mitophagy and decreased mitochondrial ROS and apoptosis caused by HG. However, the molecular mechanisms of how EPO activates PINK1/Parkin-mediated mitophagy needs to be further explored. EPO can protect kidney and other tissues by activating PI3K/Akt and AMPK/Sirt-1 signaling pathways (Wang et al., 2017; Li et al., 2019). These pathways also regulate the process of autophagy, so we will further explore whether EPO can regulate these signaling pathways to activate PINK1/Parkin-mediated mitophagy.

EPO, mainly used for treating anemia clinically, has protective effects on multiple organs and prevents tissue injury during inflammation and ischemia. Anemia has been proved to occur in the later stages of DN and increase the risk of cardiovascular diseases in patients with DN (Ritz 2006). Studies have found that EPO could prevent the increased ratio of albumin to creatinine, the loss of podocytes, and renal hypertrophy in end-stage DN mice (Loeffler et al., 2013). EPO could also reduce the production of mesangial matrix and decrease the level of oxidative stress as well as apoptosis in kidney tissues in the DN model (Jwad et al., 2018). However, these studies found that EPO had no effects on the FBG in the DN model. In our study, EPO could not only improve the morphological structure and oxidative stress of the kidney tissues of Model mice, reduce the level of mitochondrial ROS and apoptosis of mesangial cells exposed to HG, but also could reduce the FBG of Model mice significantly.

A study showed that EPO improved the loss of mitochondrial membrane potential, the increased mitochondrial ROS, and abnormal mitochondrial biosynthesis (Cui et al., 2017). In DM mice, EPO could also improve aberrant mitochondrial morphology such as mitochondrial swelling (Zhu et al., 2008). In addition, studies have shown that EPO improved the blockage of autophagy flux and activated autophagy to protect kidney tissue (Ritz, 2006; Song et al., 2015; Shi et al., 2018; Li et al., 2019). In this study, we found that EPO increased the protein expression of PINK1 and Parkin, and enhanced PINK1/Parkin-mediated mitophagy in the kidney tissues of Model mice. Also, EPO improved the excessive mitochondrial fission, the loss of mitochondrial membrane potential and increased mitochondrial ROS in mesangial cells exposed to HG. Furthermore, EPO significantly increased the ratio of LC3-II/LC3-I, the expression of PINK1 and Parkin, and significantly reduced the expression of P62, thereby improving the blockage of autophagy flux. In addition, EPO increased the number of GFP-LC3 puncta by the transfection of GFP-LC3 lentivirus, and increased the co-localization of LC3 and Parkin with mitochondria. All these results demonstrated that EPO activated PINK1/Parkin-mediated mitophagy and mitigated DN.

This study still has some limitations needed to be improved. Firstly, endogenous EPO can be mainly secreted from renal interstitial fibroblasts in the kidney (Rainville et al., 2016). Therefore endogenous EPO, which might be generated in the DN model mice, may affect the effect of exogenous EPO treatment on DN to some extent. However, several studies have demonstrated that EPO deficiency was observed in patients suffering from chronic kidney diseases and could indicate the onset of kidney diseases in diabetic patients (Mercadal et al., 2012; Fujita et al., 2019). Therefore deficient endogenous EPO may not be able to have an impact on the exogenous EPO treatment on DN. Secondly, although the expressions of mitophagy related genes in the whole lysate of renal tissues or mesangial cells displayed significant changes, it is better to also examine these expressions in the cytoplasm and mitochondria respectively. And also the co-localization evaluation of Parkin and the lysosome may help to further indicate the progression of PINK1/Parkin-mediated mitophagy. Then specific molecular mechanisms by which EPO activates PINK1/Parkin-mediated mitophagy may need to be further explored. Also, we will proceed to investigate the underlying mechanisms. Nowadays EPO is mainly used for the treatment of anemia. Due to the limited effects of medications for DN, EPO receptor agonists, which could be developed to mimic the effect of EPO, or EPO may assist to treat DN together with other medications according to the results of this study.

In conclusion, the current study showed that EPO may alleviate DN by activating PINK1/Parkin-mediated mitophagy, decreasing the level of oxidative stress and restoring the function and morphology of kidney tissues of mice in the Model group. In addition, EPO may protect glomerular mesangial cells exposed to HG by improving mitochondria function and morphology, activating PINK1/Parkin-mediated mitophagy and reducing the level of mitochondrial ROS and apoptosis.

## Data Availability

The original contributions presented in the study are included in the article/Supplementary Material, further inquiries can be directed to the corresponding authors.
