# Correction: Erythropoietin mitigates diabetic nephropathy by restoring PINK1/Parkin-mediated mitophagy

**DOI:** 10.3389/fphar.2026.1781741

**Published:** 2026-03-04

**Authors:** Xinyao Yi, Wenhui Yan, Tingli Guo, Na Liu, Zhuanzhuan Wang, Jia Shang, Xiaotong Wei, Xin Cui, Yuzhuo Sun, Shuting Ren, Lina Chen

**Affiliations:** 1 Department of Pharmacology, School of Basic Medical Sciences, Health Science Center, Xi’an Jiaotong University, Xi’an, China; 2 Department of Pathology, School of Basic Medical Sciences, Health Science Center, Xi’an Jiaotong University, Xi’an, China; 3 Key Laboratory of Environment and Genes Related to Diseases, Ministry of Education, Xi’an Jiaotong University, Xi’an, China

**Keywords:** EPO, Pink1/Parkin, LC3, mitophagy, diabetic nephropathy

There was a mistake in Figure 6 as published. In Figure 6, the GAPDH band shown in panels E and F, as well as GAPDH in panels H and J, are identical. This is because the representative images of target proteins were obtained from the same batch of gels, and therefore the corresponding GAPDH controls were displayed. However, due to a layout error, the reuse of these internal controls was not clearly indicated, which may have appeared as repeated patterns during review. The corrected figure does not affect the results and conclusions.

There was a mistake in the caption of Figure 6 as published due to the errors of panels E, F, H and J in Figure 6. The corrected caption of Figure 6 is “EPO activated PINK1/Parkin-mediated mitophagy in kidney tissues of DN mice. The changes of (A) LC3 mRNA expression, (B) P62 mRNA expression, (C) PINK1 mRNA expression, (D) Parkin mRNA expression, (E) the ratio of LC3B-II/LC3B-I, P62 protein expression and PINK1 protein expression, (G) Parkin protein expression, Drp-1 protein expression and Mfn-2 protein expression. (F) Relative quantification of LC3B-II/LC3B-I, P62 and PINK1, (H) Relative quantification of Parkin, Drp-1 and Mfn-2. Values are presented as mean ± SEM, n = 3. ^*^ *P* < 0.05, ^**^ *p* < 0.01 vs. Control group; ^#^ *p* < 0.05, ^##^ *p* < 0.01 vs. Model group.”

The corrected Figure 6 and its caption appear below.

**FIGURE 6:**
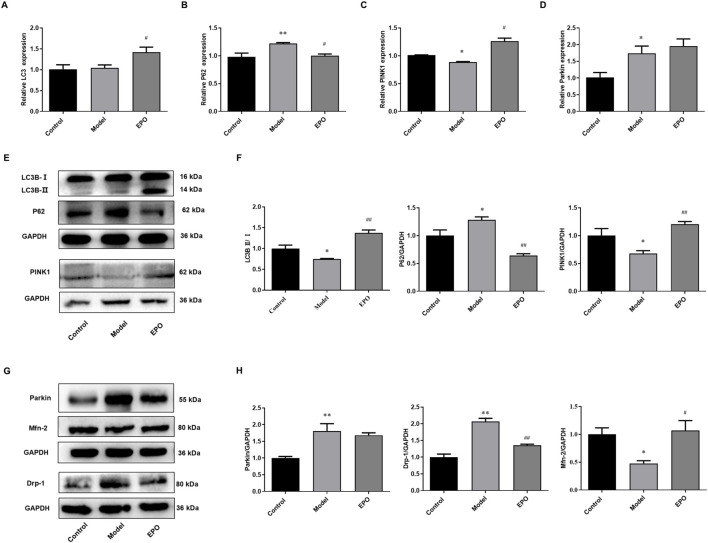
EPO activated PINK1/Parkin-mediated mitophagy in kidney tissues of DN mice. The changes of **(A)** LC3 mRNA expression, **(B)** P62 mRNA expression, **(C)** PINK1 mRNA expression, **(D)** Parkin mRNA expression, **(E)** the ratio of LC3B-II/LC3B-I, P62 protein expression and PINK1 protein expression, **(G)** Parkin protein expression, Drp-1 protein expression and Mfn-2 protein expression. **(F)** Relative quantification of LC3B-II/LC3B-I, P62 and PINK1, **(H)** Relative quantification of Parkin, Drp-1 and Mfn-2. Values are presented as mean ± SEM, n = 3. ^*^ *P* < 0.05, ^**^ *p* < 0.01 vs. Control group; ^#^ *p* < 0.05, ^##^ *p* < 0.01 vs. Model group.

Due to the correction of Figure 6, the related figure information in the main text of the paper also needed to be adjusted. A correction has been made to the section Erythropoietin Restored PINK1/Parkin-Mediated Mitophagy in Kidney Tissues of Diabetic Nephropathy Mice, paragraph 1:

“According to the *in vitro* results, the protective effects of EPO on mesangial cells exposed to HG were associated with PINK1/Parkin-mediated mitophagy. So we further detected the mRNA and protein levels of genes in PINK1/Parkin-mediated mitophagy in the kidney tissues. Compared with mice in the control group, the ratio of LC3B-II/LC3B-I (Figures 6E,F), the mRNA and protein levels of PINK1 (Figures 6C,E,F), and Mfn-2 protein level (Figures 6G,H) in the kidney tissues of mice of Model group were significantly decreased, while P62 and Drp-1 were significantly increased in terms of the mRNA and protein levels (Figures 6B,E–H), resulting in the blockage of autophagic flux and inhibited level of PINK1/Parkin-mediated mitophagy. However, there was no difference in the mRNA expression of LC3 in these two groups (Figure 6A), and the mRNA and protein levels of Parkin were elevated in the model group (Figures 6D,G,H). Of note, EPO administration partly reversed these changes as indicated by the significantly increased ratio of LC3B-II/LC3B-I (Figures 6E,F) as well as expressions of PINK1 (Figures 6C,E,F) and Mfn-2 (Figures 6G,H), and decreased expressions of P62 (Figures 6B,E,F) and Drp-1 (Figures 6G,H). These results suggested that EPO could mitigate the blockage of autophagic flux and improve the level of PINK1/Parkin-mediated mitophagy.”

The original article has been updated.

